# Picturing donations: Do images influence conservation fundraising?

**DOI:** 10.1371/journal.pone.0251882

**Published:** 2021-06-04

**Authors:** Gabby Salazar, João Neves, Vasco Alves, Bruno Silva, Diogo Veríssimo

**Affiliations:** 1 School of Forest, Fisheries, and Geomatics Sciences, University of Florida, Gainesville, Florida, United States of America; 2 Department of Science and Education, Zoomarine, Algarve, Albufeira, Portugal; 3 Department of Zoology, University of Oxford, Oxford, England, United Kingdom; Universidad Loyola Andalucia Cordoba, SPAIN

## Abstract

Many environmental organizations use photographic images to engage donors and supporters. While images play a role in fundraising, visual framing remains understudied in the environmental field. Few real-world experiments have examined which types of images result in higher donations to biodiversity conservation. We examined the role of images in conservation fundraising through a public experiment at Zoomarine, a marine park located in southern Portugal. Zoomarine runs a program called Dolphin Emotions where visitors pay to learn about dolphin biology and to interact with dolphins. We placed a donation box and a large informational poster about the Marine Megafauna Foundation, a conservation partner, in the lounge of the Dolphin Emotions program, which is open to participants and their families. The text on the poster, which solicited donations for the Marine Megafauna Foundation, was held constant, while four different image conditions were tested: dolphins, ocean wildlife, children, and people staring out from the poster (i.e., “watching eyes”). Each image condition was displayed for three days at a time and was on display for at least seven randomly assigned three-day periods over the course of 91 days. 20,944 visitors passed the donation box and the four poster conditions during this time and a total of € 952.40 was collected. The differences in mean donations in € per visitor per 3-day period were not statistically significant, *F*(3, 25) = 0.745, *p* = 0.54. Thus, we did not find that different images had a significant influence on donations to conservation. This may be due to our choice of visual frames or to the use of a donation box, which is a passive fundraising channel. Future research should examine how visual framing influences donations in other public settings and should test the influence of other visual frames on philanthropic behavior.

## Introduction

Many environmental non-governmental organizations rely on public donations to fund their conservation efforts [1, 2]. To engage supporters and elicit donations, these organizations employ a variety of different marketing techniques, including the use of powerful visual images [3, 4]. However, many marketing strategies are based on anecdotal evidence rather than empirical evidence [5, 6] and few real-world experiments have examined the impacts of environmental marketing on donation behavior [7]. To better understand donor behavior, it is important to assess real-world fundraising campaigns using rigorous methods.

Photographic images are frequently used in fundraising campaigns for environmental causes and are generally regarded as having an impact on donor behavior [8–11]. However, little is currently known about which types of images are most effective at communicating conservation messages and engaging donor support. In a recent scoping review, only 38 articles on the use of animal imagery in conservation were identified in the literature [12]. Even fewer studies examine the links between environmental images, environmental concern, and pro-environmental behaviors [12–14].

Framing theory, which helps explain how the way an issue is presented influences how people respond to that issue, can be used to study people’s responses to different types of images [15, 16]. By emphasizing certain aspects of an issue in images (e.g., showing impacts on people or on wildlife) or by visually illustrating the issue in terms of positive or negative outcomes, it is possible to shift people’s perceptions of the issue, and, in some cases, their actions [16, 17]. Although framing effects are well-documented in text, visual framing effects remain understudied [16], and have mostly been examined in relation to climate change in an environmental context [16, 17]. This gap is important because images may be more effective than text at conveying intended messages and at retaining attention [18, 19].

It is possible that certain visual frames could increase pro-social behaviors, including monetary donations to conservation. Certain visual cues may influence charitable giving and pro-social behavior, including images of charismatic flagship species [11], images of children [20], and images of watching eyes [21]. Furthermore, people may respond differently to visual appeals based on their value orientations. The Value-Belief-Norm Theory of Pro-Environmental Behavior, which been used to explain the drivers of pro-environmental behaviors, suggests that a person’s underlying values inform their beliefs about an environmental issue and whether they ultimately change their behavior [22, 23]. People with stronger altruistic values may be more concerned about environmental issues when they impact other people, while people with stronger biospheric values may be more concerned when issues impact wildlife and ecosystems [22]. Visual appeals could therefore be framed to appeal to people with different value orientations. To our knowledge, these visual frames have not been tested against each other in the conservation context.

In this study, we sought to address these gaps by running a real-world experiment at a marine park in Portugal to test the influence of different visual frames on charitable donations to a conservation project. We used a randomized controlled trial to test four different visual framing conditions paired with a donation box. We measured actual donations and compared the donations received for each visual framing condition.

## Materials and methods

**Fig 1:**
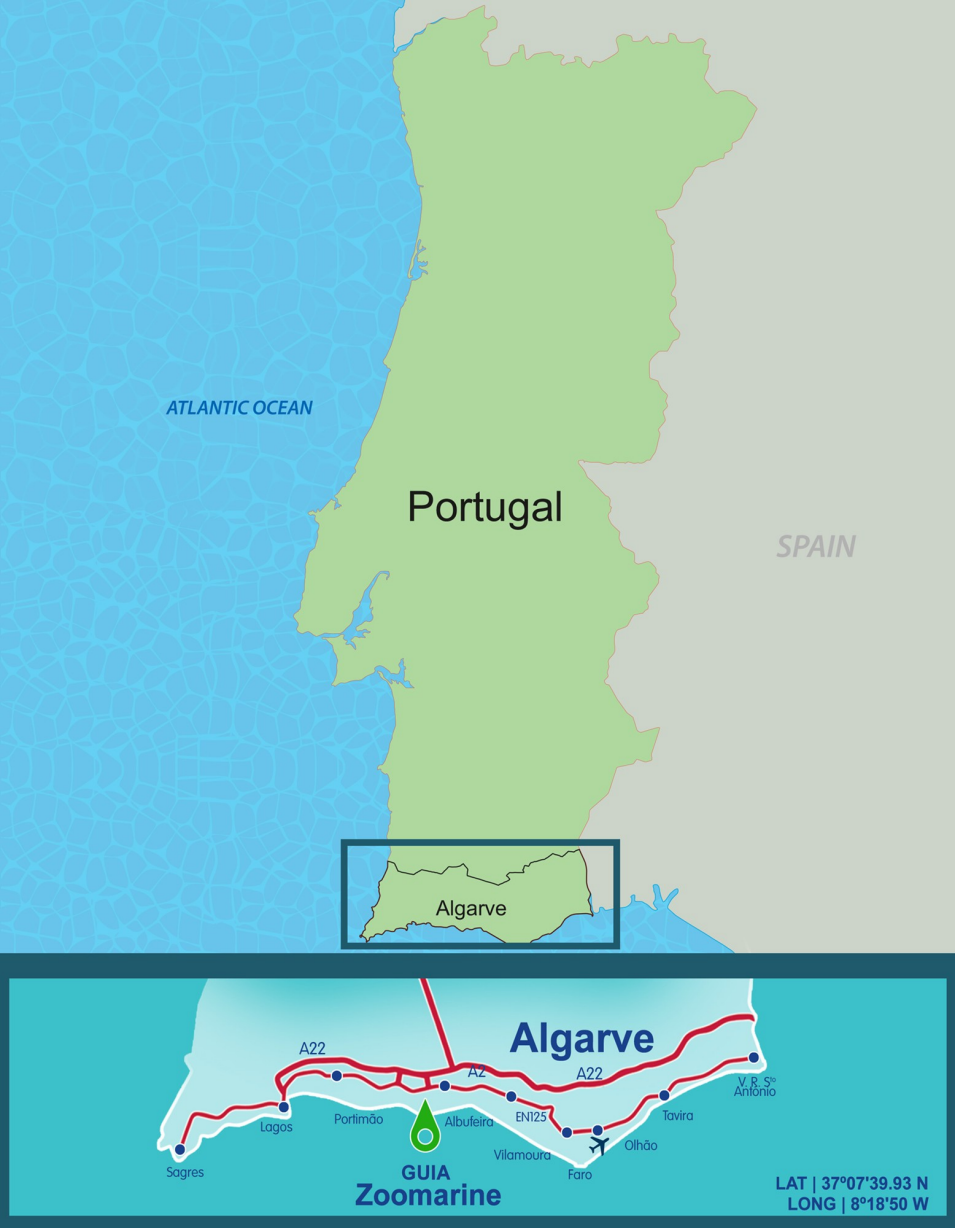
Map of Portugal, showing where Zoomarine is located.

The experiment took place at Zoomarine, a marine park located in southern Portugal (Fig 1). Zoomarine runs an education program called Dolphin Emotions where visitors pay an additional fee to learn about dolphin biology and ecology and to interact with dolphins. This study was approved by the University of Florida’s Institutional Review Board (IRB202002372). Consent was not obtained because data were collected and analyzed anonymously.

**Fig 2:**
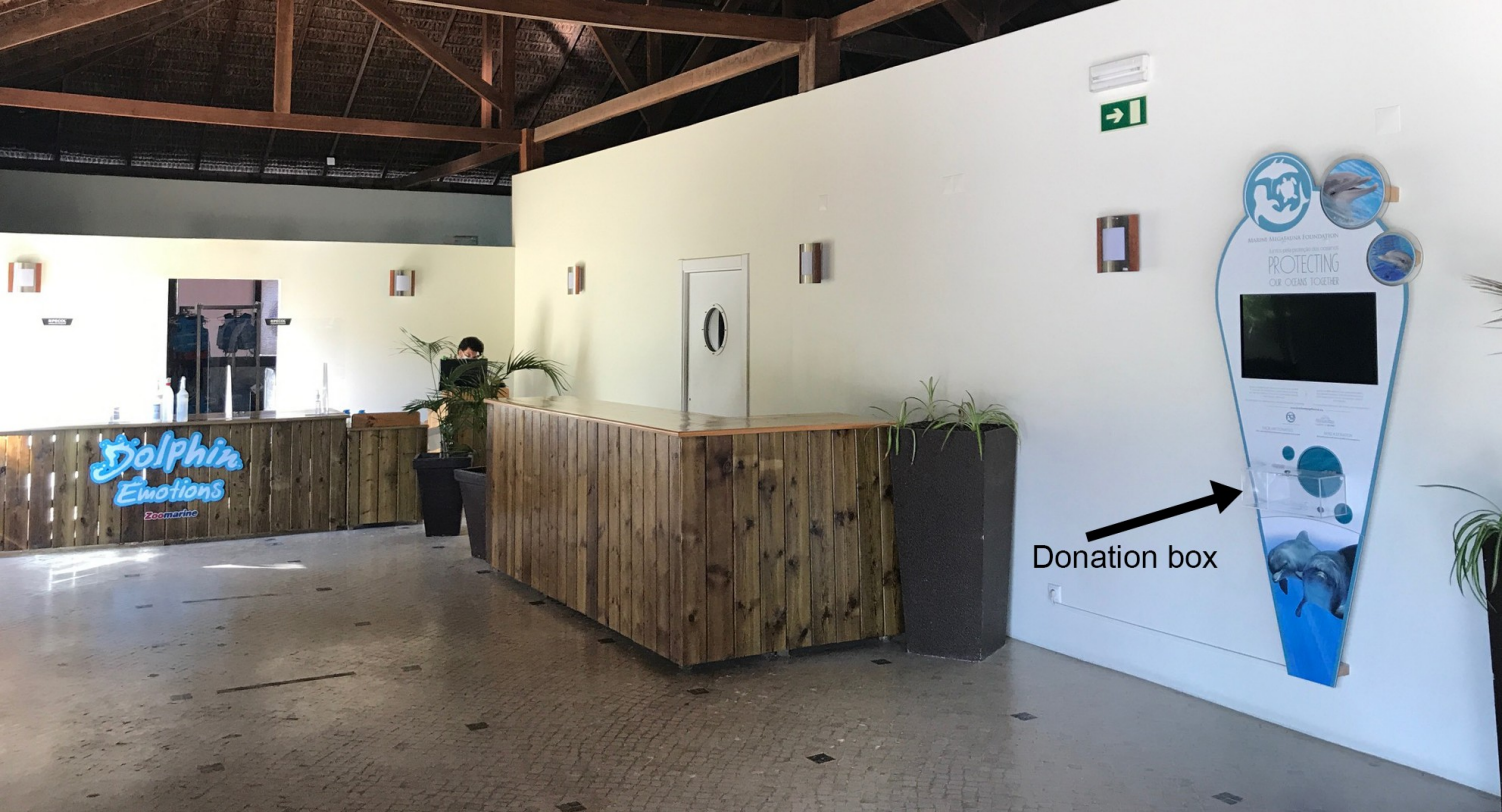
An image of the Dolphin Emotions lounge. One of the poster conditions is seen with the transparent donation box (right).

For this study, a transparent donation box and large informational poster (207 x 90 cm) were placed in the public lounge of the Dolphin Emotions program, which only program participants and their families are able to access (Fig 2). The poster framed a small video screen where a short video about a conservation group, the Marine Megafauna Foundation, played consistently on a loop. Participants pass through the lounge twice during the program, both on their way to participate in the Dolphin Emotions program and as they exit the experience. This enabled us to track the number of visitors who passed by the donation box. While demographic details were not collected about all Dolphin Emotions program participants during this period, around 2200 participants completed a satisfaction survey that collected some demographic data. From this survey, we know that 63% of these visitors identified as female and 37% identified as male and that 45% were Portuguese and 55% were from other countries. Participants came from a range of age groups, although around 90% of participants were under 50 years of age and many were children or adolescents (47% of female participants and 44% of male participants were between 8–17 years old). Many of these children and adolescents would have been accompanied by older family members since the average size of groups, including participants and their relatives, was four individuals. While we do not have data on whether visitors participated in the program multiple times during the study period, it is unlikely that there were repeat visitors given the financial cost involved in visiting Zoomarine and the additional cost of participating in the Dolphin Emotions program. The individual in Fig 2 is an employee, not a study participant, and has given written informed consent (as outlined in PLOS consent form) to be pictured in this manuscript.

**Fig 3:**
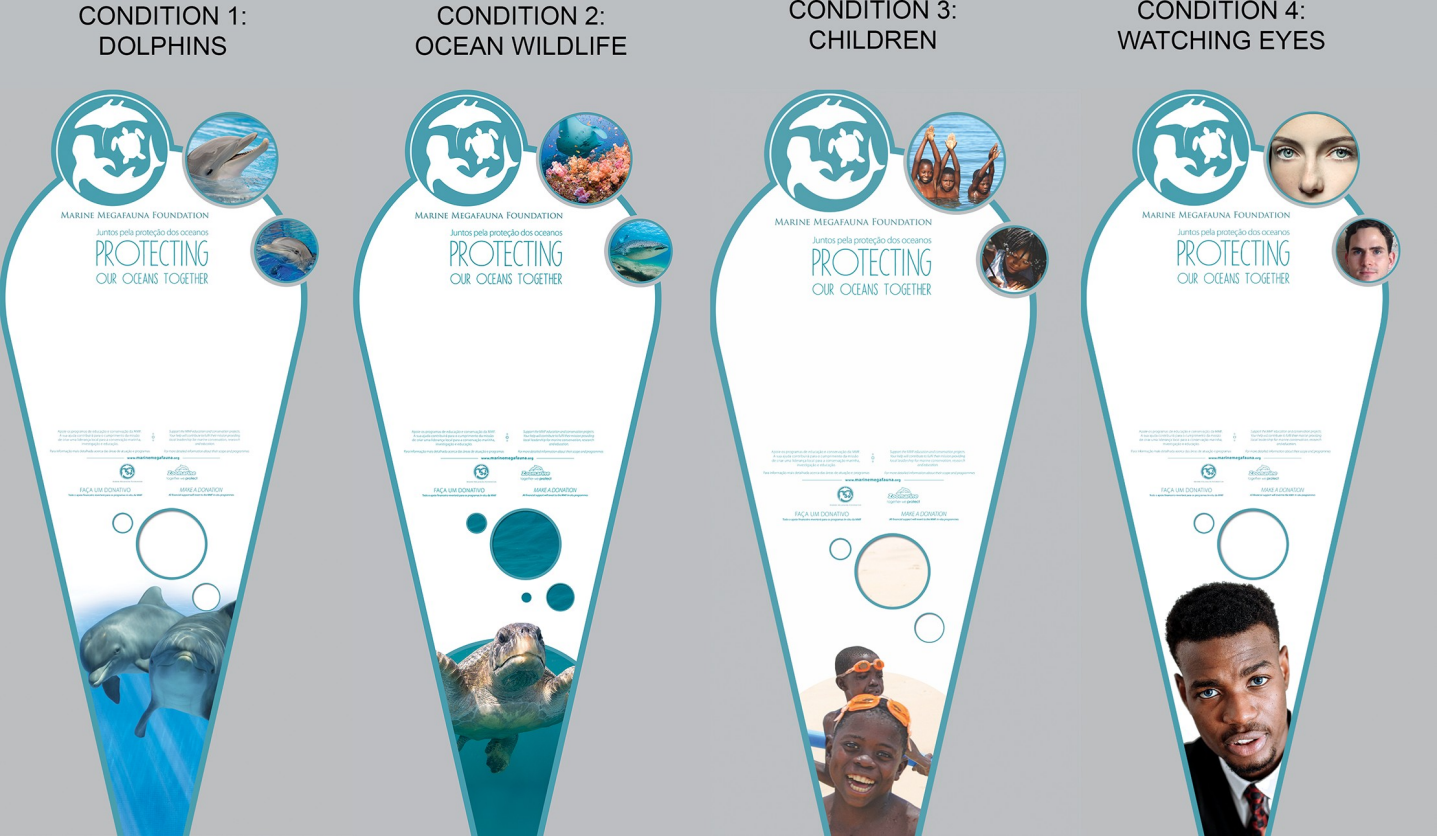
The four poster conditions that were tested in this study, paired with a clear donation box. This figure is illustrative; some of the exact images used in this figure have been changed due to licensing restrictions.

The experiment ran for a total of 91 days between June 28 and September 26, 2019, although data were only collected for 87 days due to park closures on four days. The text displayed on the poster (which was in both English and Portuguese) and the video playing on the screen related to the ocean conservation work of the Marine Megafauna Foundation, a partner of Zoomarine, and was held constant throughout the experiment. The short video played on a loop on the screen and highlighted the positive efforts of the organization to conserve ocean wildlife. The board clearly stated that all collected funds would be given to the Marine Megafauna Foundation. Four poster conditions were tested, differing only in terms of the images presented (Fig 3). Each poster condition was displayed for three days at a time and was on display for at least seven three-day periods during the experiment. Poster conditions were randomly assigned to different three-day periods over the course of 91 days. Additionally, to ensure that treatment effects were not confounded by day-of-week effects, the experimental conditions were randomized across time, ensuring that all conditions had equal probability of being displayed on any given day of the week (S1 Table).

### Condition 1: Dolphins (biospheric value appeal)

The first condition displayed images of dolphins, the flagship species of the Dolphin Emotions program and a species that is thought to be highly charismatic [24]. We used this as a baseline condition because single charismatic flagship species are commonly used in fundraising appeals by environmental organizations [25]. Our goal was to see whether other conditions could improve upon this baseline. This condition was likely to appeal to people with strong biospheric values and was also closely aligned with the objectives of the Dolphin Emotions program and the conservation program featured on the poster. Appeals with images of flagship species have been shown to result in higher donations to conservation than appeals with images of non-flagship species [11].

### Condition 2: Ocean wildlife (biospheric value appeal)

The second condition used images of multiple charismatic flagship species from the ocean (a sea turtle, a manta ray, and a whale shark) to test whether the use of a flagship fleet, or a group of flagship species, was more effective than a single flagship species [26]. This condition was also likely to appeal to people with strong biospheric values.

### Condition 3: Children (altruistic value appeal)

The third condition included images of happy children to test whether framing the issue in terms of its influence on humans would be more effective. Altruism is a strong motivator for charitable giving [27, 28] and images of children in charitable advertising have been found to provoke stronger emotional reactions than images of adults [20].

### Condition 4: Watching eyes

The fourth condition included images of people staring out from the poster to test the “watching eyes” hypothesis, which suggests the feeling of being watched by eyes that are directly gazing at the viewer may increase the likelihood that individuals engage in pro-social behaviors, such as charitable giving [29, 30]. Field experiments have demonstrated that the presence of watching eyes increases the number of people who pay for their drinks using an honesty box [31], who donate to charity [21], and who pick up litter [32].

At the beginning of each three-day period, the same amount of seed money (€ 100) was put in the box before visitors arrived to encourage further donations. At the end of each three-day treatment period, the amount of money collected was withdrawn from the donation box and counted.

## Results

There were four conditions, each in place for at least seven three-day periods: three-day periods with images of dolphins condition (*n* = 7), three-day periods with images of ocean wildlife condition (*n* = 7), three-day periods with images of children condition (*n* = 8), and three-day periods with images of watching eyes condition (*n* = 7). The experiment ran for 91 days and data were collected on 87 of those days. A total of 20,944 visitors passed by the donation box and the different poster conditions during this time. In total, € 952.40 were collected in the donation box with 84% of that amount being in coins with a value of €1 or less. The mean and median number of visitors per 3-day period for each poster condition were somewhat different: dolphin condition (*M* = 710; *Mdn* = 590 visitors), watching eyes condition (*M* = 753; *Mdn* = 731 visitors), ocean wildlife condition (*M* = 833; *Mdn* = 832 visitors), and children condition (*M* = 609; *Mdn* = 536 visitors). However, while there are differences between conditions, we have adjusted for this difference by taking the mean donation per visitor for each three-day period.

We found that neither a one-way ANOVA (parametric) nor a Kruskall Wallis H test (nonparametric) showed significant differences between treatments in terms of the mean donation per visitor per 3-day period. We first used a one-way ANOVA, a more conservative test, to determine if the mean donation per visitor per 3-day period (in €) was different for different poster conditions. There was homogeneity of variances, as assessed by Levene’s test for equality of variances (*p* = 0.187). There were three outliers in the data (3-day periods that had higher donations per visitor than other periods for a condition), as assessed by boxplots, but the test results were not materially affected by them, as determined by comparing the result of a one-way ANOVA with and without the outliers (S1 Appendix). Visual inspection of Normal Q-Q plots showed that data were close to normally distributed (S2 Appendix). However, while the results of a Shapiro-Wilk test demonstrated that the data were normally distributed for the watching eyes (*p* = 0.141), children (*p* = 0.469), and ocean wildlife conditions (*p* = 0.392), the data for the dolphin condition was not normally distributed (*p* = 0.037). Mean donations in € per visitor per 3-day period for each condition increased from the watching eyes condition (*M* = 0.036, *SD* = 0.023), to the children condition (*M* = 0.041, *SD* = 0.025), to the dolphins condition (*M* = 0.060, *SD* = 0.056), to the ocean wildlife condition (*M* = 0.060, *SD* = 0.046), in that order, but the differences between the different conditions were not statistically significant, *F*(3, 25) = 0.745, *p* = 0.54 (Table 1). Because the one-way ANOVA is conservative and because the data were not normally distributed for all conditions, we also conducted a Kruskall Wallis H test (nonparametric) to see if the type of test would influence our conclusions. Nonparametric tests are also more robust to outliers. We found that the distributions of donations per visitor were not similar for all treatments, as assessed by visual inspection of a boxplot. Median donations per visitor increased from eyes (0.032), to children (0.033), to dolphins (0.041), to ocean wildlife (0.041), but the differences were not statistically significant, χ^2^(3) = 1.576, *p* = 0.665.

**Table 1:** Mean donations per visitor per 3-day period by condition.

Condition	Number of 3-day periods	M (donations per visitor per 3-day period in €)	SD
Dolphins	7	0.060	0.056
Ocean Wildlife	7	0.060	0.046
Children	8	0.041	0.025
Watching Eyes	7	0.036	0.023

## Discussion

This randomized controlled trial tested whether using different visual images on a solicitation poster would influence the amount of donations placed in a donation box at a marine park in Portugal. We sought to understand whether images of children, images of watching eyes, or images of multiple charismatic flagship species could garner more donations than images of a single, charismatic flagship. We did not find that different visual frames had a significant influence on donations to conservation. Overall, the average donation per visitor was low across conditions, with less than €1000 placed in the donation box over the course of 87 days from over 20,000 visitors. The low donation per visitor could be partly explained by the number of visitors who visit the park in family units, which effectively reduces the number of potential donors.

There are a number of potential explanations for our results. First, donation boxes are a passive fundraising channel, requiring low involvement on the part of the donor and constituting a low risk to the donor [33]. Donors, therefore, may not have paid close attention to the visuals on the posters when deciding whether or not to donate. Instead, they may have been motivated to give by their general attitude toward the marine park and by their experiences there [33]. A recent study tested whether using different flagship species on donation boxes would influence monetary donations in Australia and found that only the body mass of animals had a small influence on the amount donated per customer [7]. Flagship type, appeal, and familiarity did not have a significant influence on donations. The authors suggest that donor’s attitudes toward the broader fundraising organization may have had a larger influence on donations than the differences in solicitation posters displayed across conditions [7].

Our choice of visual conditions may have also influenced results. While three of the poster conditions used images with a positive valence, none of the conditions used images with a negative valence. Some research suggests that positively framed images may inspire engagement but may also lead to complacency [8, 34]. In contrast, images with a negative valence (e.g., those illustrating losses or problems) may be more likely to inspire donations [20, 35]. In future experiments, it would be interesting to include a condition illustrating a threat to the flagship species (e.g., dolphins caught in nets). It may also be important to test whether image size has an effect on donations. Visual saliency is likely to influence attention, with larger and more salient images attracting more attention [36]. It would also be interesting to test one condition without images to see if images make a difference at all. Donation boxes paired with images have been shown to elicit more donations than donation boxes without images [37].

We did not find that images of watching eyes, or eyes that give the perception of a direct gaze, elicited more donations than other treatments. While watching eyes have been shown to increase donations to charity in some settings, results are mixed and may also be influenced by perceived norms around giving [38]. For example, an experiment found no general effect when it tested whether images of human eyes on recycling machines at supermarkets would increase the number of people who chose to donate the money they received to charity [39]. However, a field experiment at a children’s museum found that including eyes on a donation solicitation sign resulted in significantly more donations than an image of a human nose or a chair, but not a human mouth [40]. Experiments testing this effect often use close-up images of eyes, rather than images of people’s faces (e.g., [31, 40]). In our experiment, the inclusion of a face may have influenced the treatment. If perceived social norms influence pro-social behavior, the physical appearance of the person in the images may play a role in whether or not people respond to images of faces. A person might be more likely to respond if they associate the person pictured as part of their in-group, which could be influenced by gender, age, race, or ethnicity, among other factors [41, 42]. Additionally, it is important to acknowledge that all four poster treatments we tested included organisms with eyes (e.g., turtles, children, dolphins). However, only the watching eyes condition included images that focused in on people’s eyes and gave the appearance of surveillance through a direct gaze [29].

One limitation of the study is that we were unable to monitor the number of individuals that donated, which may have varied between conditions. In some cases, a few individuals clearly made large donations, which influenced results. In one three-day period when the ocean wildlife condition was on display, two €50 notes were put in the donation box, leading to a total of €140.22 in the donation box for that 3-day period. This is €73.82 more than any other three-day period in the experiment. However, as mentioned in the results, removing this outlier did not materially affect the outcome of the experiment. The vast majority of donations (84%) were in coins of €1 or less. The use of a cash-based donation box is another limitation. Other visitors may have wanted to donate (or to donate larger amounts) but may not have had cash with them. Future experiments could include a card terminal allowing for contactless payments or a text-to-give option to ensure that all visitors have the opportunity to contribute. Additionally, many visitors were likely in family units and may have made a donation as a group. This suggests that it might be interesting to calculate donation per group instead of donation per individual visitor in future studies. Finally, the video in the poster likely created tradeoffs. On the one hand, the video may have attracted people who would not have otherwise looked at the display. On the other hand, it might have competed for the attention of visitors, obscuring the effect of different poster conditions [43]. The latter could be particularly meaningful if the different visual conditions only had a small effect. However, it is unclear whether videos or images would be more salient in this context; a recent review suggests that the relative salience of video and still images appears to be context-dependent [44].

It is clear that much remains to be understood when it comes to the relationship between visual marketing and charitable giving. Future research should examine how visual framing influences donation behavior in public settings and should test these effects outside of animal-oriented settings, such as zoos and marine parks, to see if the other public audiences respond differently to visual frames. These insights may well reveal themselves to be critical to the effectiveness and efficiency of fundraising for biodiversity conservation.

## Supporting information

S1 Fig (PDF)

S1 Table Schedule of conditions displayed. (DOCX)

S1 Appendix One-way ANOVA test results with and without outliers. (DOCX)

S2 Appendix Normal Q-Q plots for each treatment. (DOCX)
