# Supplementary material for: Picturing donations: Do images influence conservation fundraising?
Source: PLoS One. 2021 Jun 4;16(6):e0251882. doi: 10.1371/journal.pone.0251882 (PMC8177415; doi:10.1371/journal.pone.0251882)
Supplement: S1 Table — (DOCX) [file pone.0251882.s002.docx]

**S1 Table. Schedule of conditions displayed.**

To avoid the possible bias of specific weekdays (e.g., having more visitors on certain days), the experimental conditions were randomized across time, ensuring that all conditions had equal probability of being displayed on any given day of the week. This is the resulting schedule. Note: Starting in mid-September, the park was closed on Mondays and Tuesdays.

| Timeline | Day at start of 3-day period | Condition |  |  |  |
| --- | --- | --- | --- | --- | --- |
| 6/28/19 | Friday | Ocean Wildlife |  |  |  |
| 7/1/19 | Monday | Children |  | Condition | Number of 3-day periods |
| 7/4/19 | Thursday | Watching Eyes |  | Dolphins | 7 |
| 7/7/19 | Sunday | Children |  | Watching Eyes | 7 |
| 7/10/19 | Wednesday | Watching Eyes |  | Ocean Wildlife | 7 |
| 7/13/19 | Saturday | Dolphins |  | Children | 8 |
| 7/16/19 | Tuesday | Watching Eyes |  |  |  |
| 7/19/19 | Friday | Children |  |  |  |
| 7/22/19 | Monday | Children |  |  |  |
| 7/25/19 | Thursday | Ocean Wildlife |  |  |  |
| 7/28/19 | Sunday | Dolphins |  |  |  |
| 7/31/19 | Wednesday | Children |  |  |  |
| 8/3/19 | Saturday | Ocean Wildlife |  |  |  |
| 8/6/19 | Tuesday | Ocean Wildlife |  |  |  |
| 8/9/19 | Friday | Ocean Wildlife |  |  |  |
| 8/12/19 | Monday | Dolphins |  |  |  |
| 8/15/19 | Thursday | Dolphins |  |  |  |
| 8/18/19 | Sunday | Watching Eyes |  |  |  |
| 8/21/19 | Wednesday | Watching Eyes |  |  |  |
| 8/24/19 | Saturday | Watching Eyes |  |  |  |
| 8/27/19 | Tuesday | Ocean Wildlife |  |  |  |
| 8/30/19 | Friday | Children |  |  |  |
| 9/2/19 | Monday | Dolphins |  |  |  |
| 9/5/19 | Thursday | Ocean Wildlife |  |  |  |
| 9/8/19 | Sunday | Dolphins |  |  |  |
| 9/11/19 | Wednesday | Children |  |  |  |
| 9/14/19 | Saturday | Watching Eyes |  |  |  |
| 9/19/19 | Thursday | Children |  |  |  |
| 9/22/19 | Sunday | Dolphins |  |  |  |
