# Supplementary material for: Picturing donations: Do images influence conservation fundraising?
Source: PLoS One. 2021 Jun 4;16(6):e0251882. doi: 10.1371/journal.pone.0251882 (PMC8177415; doi:10.1371/journal.pone.0251882)
Supplement: S1 Appendix — (DOCX) [file pone.0251882.s003.docx]

**S1 Appendix: One-way ANOVA test results with and without outliers.**

We conducted a one-way ANOVA with and without the three outliers in the dataset (3-day periods that had higher donations per visitor than other periods for a condition). The following results demonstrate that the results were not materially affected by the outliers, as determined by comparing the results of a one-way ANOVA with and without the outliers.

Results of one-way ANOVA with the three outliers included: *F*(3, 25) = 0.745, *p* = 0.54

Results of one-way ANOVA with the three outliers removed: *F*(3, 22) = 0.676, *p* = 0.58
