# Supplementary material for: Picturing donations: Do images influence conservation fundraising?
Source: PLoS One. 2021 Jun 4;16(6):e0251882. doi: 10.1371/journal.pone.0251882 (PMC8177415; doi:10.1371/journal.pone.0251882)

**S2 Appendix**: Normal Q-Q Plots for each treatment. These plots were used to check whether data were normally distributed (A = dolphins; B = watching eyes; C = ocean wildlife; D = children).

A

B
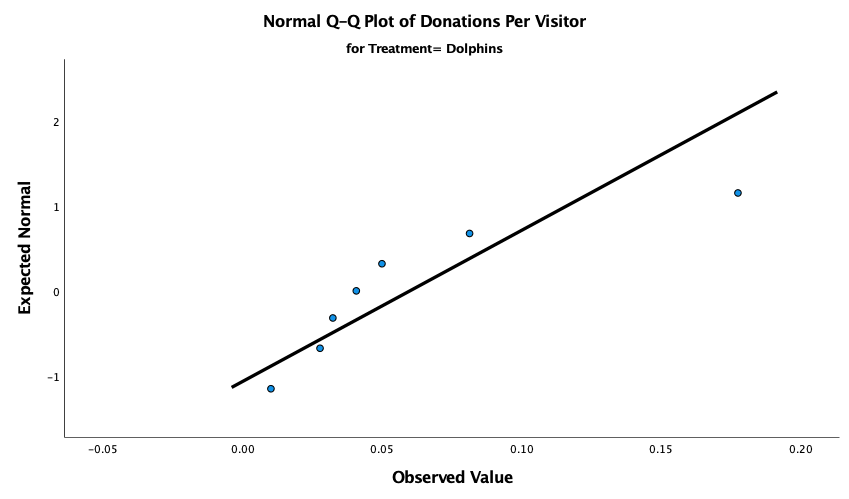


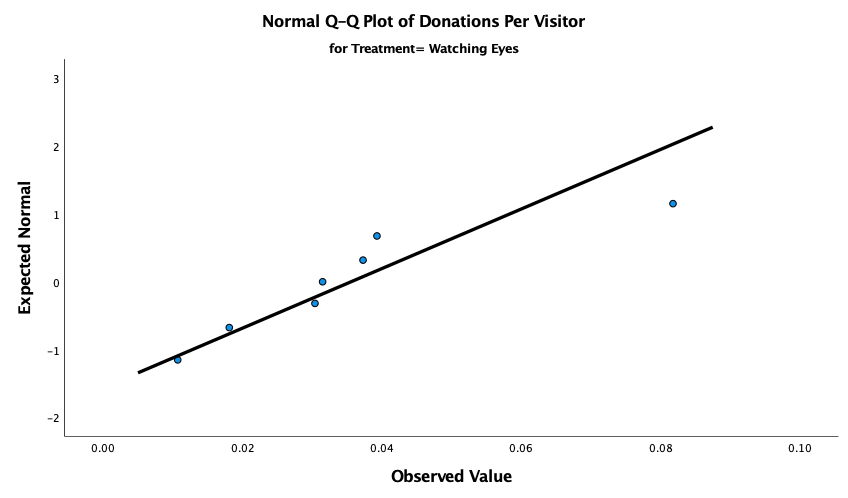


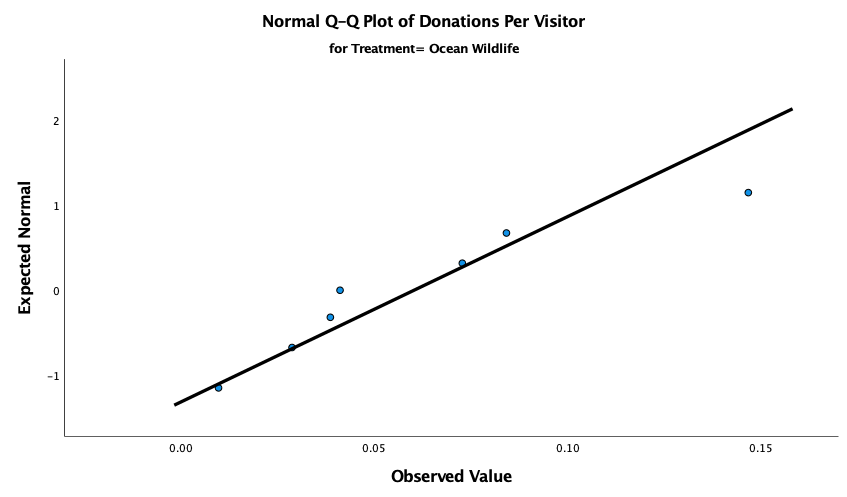


D


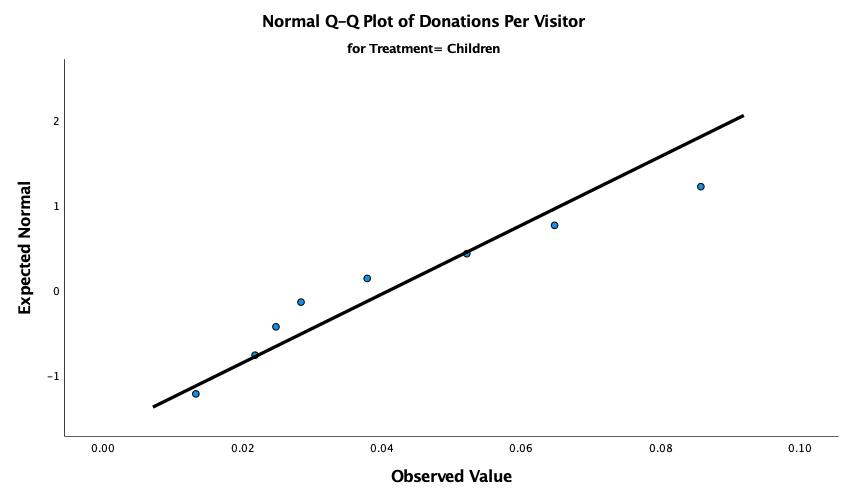

Supplement: S2 Appendix — (DOCX) [file pone.0251882.s004.docx]
